# Comparison and Assessment of Anti‐Inflammatory and Antioxidant Capacity Between EGCG and Phosphatidylcholine‐Encapsulated EGCG

**DOI:** 10.1111/jocd.16628

**Published:** 2024-10-31

**Authors:** Minjia Yuan, Lili Hu, Cuicui Zhu, Qi Li, Hang Tie, Haihua Ruan, Tao Wu, Hongyang Zhang, Liang Xu

**Affiliations:** ^1^ School of Pharmacy Tianjin Medical University Tianjin People's Republic of China; ^2^ Shanghai Qiran Biotechnology Co. Ltd. Shanghai People's Republic of China; ^3^ Shanghai Jinjia Technology Co. Ltd. Shanghai People's Republic of China; ^4^ Chinese Academy of Inspection & Quarantine Greater Bay Area Guangdong People's Republic of China; ^5^ Tianjin Key Laboratory of Food Biotechnology College of Biotechnology and Food Science, Tianjin University of Commerce Tianjin China

**Keywords:** antioxidant, cosmetic raw materials, EGCG, phosphatidylcholine‐encapsulated EGCG, proinflammatory factors

## Abstract

**Aim:**

To compare and evaluate the differences between EGCG and phosphatidylcholine‐encapsulated EGCG in terms of their anti‐inflammatory and antioxidant capacities.

**Methods:**

In this study, transdermal absorption experiments were conducted to compare the absorption capacity of EGCG and phosphatidylcholine‐encapsulated EGCG. Subsequently, the disparity in anti‐inflammatory and antioxidant efficacy between EGCG and phosphatidylcholine‐encapsulated EGCG were evaluated through cytotoxicity experiments, as well as the determination of cellular inflammatory factors and the measurement of ROS content under different treatment conditions.

**Results:**

The concentration of EGCG, encapsulated in phosphatidylcholine, in porcine skin is 40.76 ± 1.29 μg/cm^2^, which is significantly higher than the concentration of EGCG alone (31.62 ± 2.01 μg/cm^2^). Also, the ability of phosphatidylcholine‐encapsulated EGCG to suppress inflammatory factors such as tumor necrosis factor‐α (TNF‐α), cyclooxygenase‐2 (COX‐2), and prostaglandin E2 (PGE2) was notably superior to that of EGCG alone. Both phosphatidylcholine‐encapsulated EGCG and EGCG showed excellent ROS scavenging ability in terms of antioxidant capacity.

**Conclusion:**

The percutaneous absorption and anti‐inflammatory impact of EGCG encapsulated within phosphatidylcholine were substantially enhanced when compared to EGCG by itself. Additionally, both formulations exhibited enhanced ROS scavenging capacities, albeit the variance between them was not pronounced. These insights furnish a vital theoretical underpinning for the utilization of phosphatidylcholine‐encapsulated EGCG in cosmetic applications, specifically for fostering products with anti‐inflammatory and antioxidant benefits.

## 1 Introduction

The global cosmetics industry is experiencing substantial growth in recent years and continues to expand in the future [1]. Cosmetics ingredients can be categorized into two types: synthetic and natural materials, offering a wide range of options [2, 3]. While both types of ingredients offer effective functional benefits, it is essential to recognize that they may also pose certain risks and, in severe cases, can potentially contribute to the development of skin disorders. Among the numerous research efforts underway regarding cosmetic ingredients, a key focus is on enhancing and optimizing existing natural ingredients. This initiative holds significant importance for the development and advancement of the cosmetics industry.

Epigallocatechin gallate (EGCG) is a natural cosmetic ingredient derived from tea polyphenols. It is widely regarded as the most potent active component among catechins [4]. Extensive research has demonstrated that EGCG possesses significant properties, including antibacterial, antiviral, antioxidant, antiatherosclerotic, antithrombotic, antiangiogenic, anti‐inflammatory, and antitumor activities [5, 6, 7]. Also, as a potent antioxidant, EGCG demonstrates the ability to effectively clean up a wide range of reactive oxygen species (ROS) and reactive nitrogen species (RNS), which include superoxide anion radicals, hydrogen peroxide, hydroxyl radicals, singlet oxygen, nitric oxide, peroxynitrite, etc. These substances have been found to closely link to human diseases and have the potential to induce inflammatory responses and even carcinogenesis. EGCG possesses the ability to scavenge peroxyl radicals, thereby interrupting lipid peroxidation facilitated by free radicals [8]. Research has revealed that exposure of human skin fibroblasts (HSF) to UVA irradiation can lead to a reduction in cellular levels of superoxide dismutase (SOD) and glutathione peroxidase (GSH‐Px), resulting in an increase in malondialdehyde (MDA) activity [9]. Nevertheless, the introduction of EGCG substantially reinstates the activity of SOD and GSH‐Px, thereby diminishing MDA levels. This effectively mitigates UVA‐induced oxidative damage and significantly reduces the detrimental effects of UV radiation [10, 11]. In a study conducted by Hong Wei et al. [12], lipopolysaccharide (LPS)‐induced macrophages were treated with Cu‐EGCG nanoparticles, resulting in a remarkable enhancement in cell viability. Furthermore, these nanoparticles demonstrated a notable ability to efficiently eliminate various ROS, such as hydroxyl radicals and superoxide anions. Additionally, the application of Cu‐EGCG nanoparticles proved beneficial in improving the pathological microenvironment of inflamed synovial chondrocytes by reducing ROS levels and attenuating inflammatory responses [12].

**FIGURE 1:**
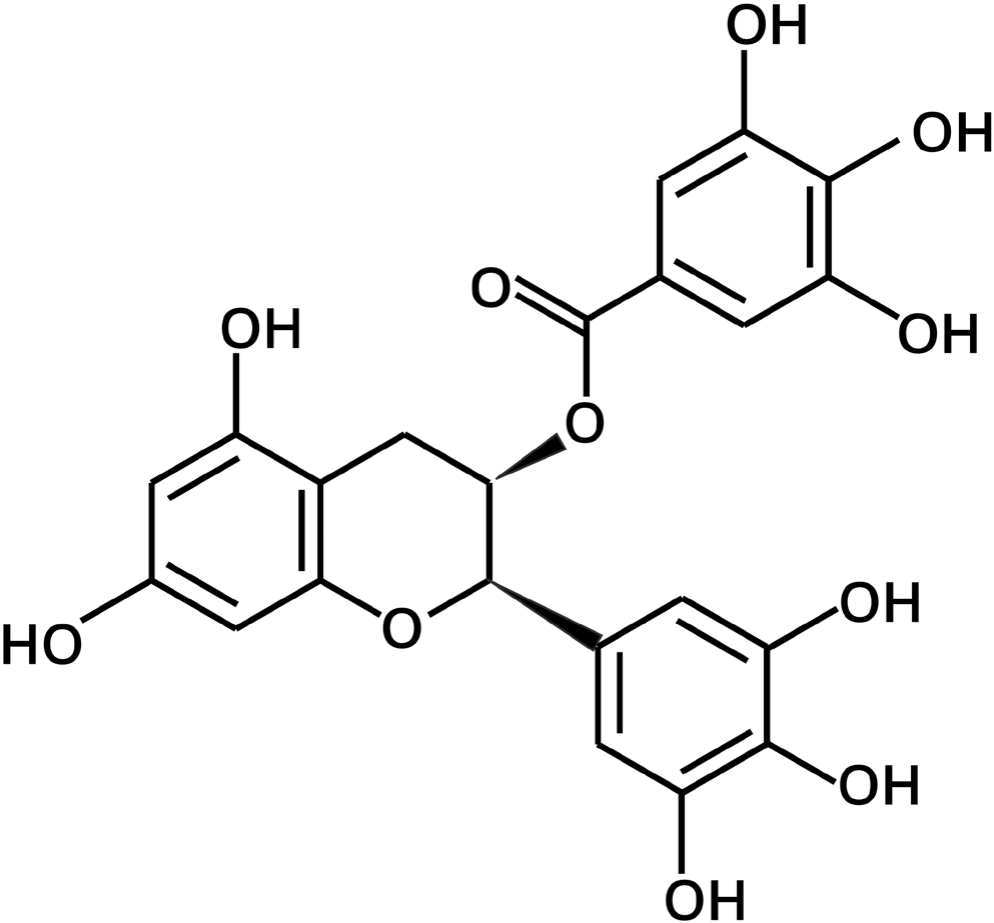
The structure of epigallocatechin gallate [13].

EGCG possesses a tetracyclic structure with eight hydroxyl groups (Figure 1). The presence of numerous hydroxyl groups enables EGCG to readily form complexes with natural macromolecules, such as polysaccharides or proteins [8]. The tetracyclic structure of EGCG contributes to its solubility in water, while also exhibiting some solubility in hydrophobic substances. It is well recognized that cell membranes primarily comprise phospholipid bilayers, with biological macromolecules like proteins and polysaccharides distributed on their surface or within their interior. When EGCG is incorporated into cosmetics, its hydrophilic nature hampers its efficient absorption by human skin cells, thereby hindering its access to target sites [8]. The modification of amphiphilic polymers with phosphatidylcholine, mimicking the structure of cell membranes, is a common strategy employed to enhance biocompatibility. This approach has found widespread use in diverse fields, such as blood purification [14], artificial organs [15], and pharmaceuticals [16]. In our study, we utilized phosphatidylcholine to encapsulate EGCG, thereby forming a complex. The objective was to improve the solubility, stability, and transdermal delivery of EGCG while preserving its therapeutic effects. Nevertheless, additional assessment is necessary to evaluate the effectiveness of EGCG encapsulated within phosphatidylcholine mimicking cell membrane coatings. The object of this study is to conduct a comparative analysis of the anti‐inflammatory potential and the antioxidant properties of EGCG and phosphatidylcholine‐encapsulated EGCG, aiming to assess the effectiveness enhancement of phosphatidylcholine encapsulation on EGCG and obtain high‐quality EGCG. Furthermore, this study tries to demonstrate the ability of phosphatidylcholine as a modification method for cosmetic ingredients to enhance their efficacy, which will provide crucial evaluation criteria and application guidelines for the utilization of phosphatidylcholine in a broader range of cosmetic ingredients.

## 2 Materials and Methods

### 2.1 Materials

The following materials were used: Trypsin (500 mL, BasalMedia); High glucose DMEM medium (500 mL, Corning); FBS (500 mL, AusGeneX); antibiotics, (500 mL, BasalMedia); CCK‐8 (50 mL, Beyotime); Mouse TNF‐α ELISA Kit (MultiSciences (Lianke) Biotech Co. Ltd.); Mouse COX‐2 ELISA Kit (Elabscience); Mouse PGE2 ELISA Kit (Lanpai Biosciences); Bama minipig skin for transdermal experiments (Huilang Baio); EGCG (Jiangsu Tiansheng Pharmaceutical Co. Ltd.); Phosphatidylcholine (Guangzhou Hanfang Pharmaceutical Co. Ltd); DCFH‐DA (JiZhi Biochemical); 3D epidermal skin model (EpiKutis, Guangdong Biocell Biotechnology, Co. Ltd.) FHC‐1200A Biological safety cabinet (ESCO Micro); BSL‐2 Cell culture clean device (ThermoFisher); GERMANY 5424R Tabletop centrifuge (EPPENDORF AG); SpectraMax M5 Microplate reader (Molecular Devices); JY300C Electrophoresis apparatus (Beijing Junyi Dongfang); Diffusion pools (Huilang Baio); M165FC fluorescence microscope (Leica Camera AG); UVB ultraviolet lamp (Yixian); Cell counter (Count star).

### 2.2 The Required Cells and Culture Conditions

The cells used for this study were as follows: mouse monocyte/macrophage leukemia cells (Raw264.7) and human keratinocytes (HaCaT). The cells were cultured in DMEM medium containing 10% fetal bovine serum and 1% antibiotics. The cultures were maintained at 37°C in a 5% CO_2_ incubator and the medium was changed every 48 h (adjustments could be made based on cell growth). Passaging was performed when the cell concentration reached 80%–90%. The culture medium was aspirated, and the cells were washed with PBS buffer two to three times. Then, 1 mL of 0.25% trypsin was added for 30 s (the digestion time may vary depending on the cell condition), and the reaction was stopped by adding 2 mL of culture medium. The cells were centrifuged at 800 rpm for 5 min, and 10 mL of culture medium was added for subsequent passages at a ratio of 1:2–1:3. Experiments were conducted using cells from the third to eighth passages [17, 18].

### 2.3 Preparation of Phosphatidylcholine‐Encapsulated EGCG

EGCG (0.8 g) was dispersed in 80 mL of 0.2 M phosphate buffer (pH 7.0). Phosphatidylcholine (20 g) was then added, and the dispersions were maintained in a water bath at 80°C for 1 h. Subsequently, 68 mL of phosphate buffer and 12 mL of glycerol were added, and the mixtures were again kept at 80°C for 1 h. The suspension volume was adjusted to a final volume of 240 mL with phosphate buffer. The samples were vortexed at 60°C and then sonicated at a measured power of 120 W for 5 min, with a 60‐s pause every minute to allow for cooling. The resulting samples were freeze‐dried and stored at −20°C. A sample of phosphatidylcholine without EGCG was also prepared using this method and used as a blank control for subsequent study. The model of the phosphatidylcholine‐encapsulated EGCG that was prepared is illustrated in Figure S1.

### 2.4 Cytotoxicity Assay

Cells were seeded at a density of 5 × 10^4^ cells/mL in a 96‐well plate and cultured for 12–24 h (adjustments can be made based on cell growth). Different concentrations of the test drug (10 μL) were added to each well of the 96‐well cell culture plate. The plate was then incubated in a CO_2_ incubator for 24 h. After 24 h of treatment with different concentrations of the samples, the culture medium was aspirated, and the cells were washed with 100 μL of PBS. The PBS was then removed, and 100 μL of cell culture medium containing 10% CCK‐8 was added to each well. After incubating for 1–4 h (adjustments can be made based on the CCK‐8 staining), the absorbance at a wavelength of 450 nm was measured using a microplate reader to calculate the cell viability [19, 20]. The calculation formula was as follows: Cell viability = [(A_s_—A_b_)/(A_c_—A_b_)] × 100%, where A_s_ represents the absorbance of the experimental well (containing cells, culture medium, CCK‐8 solution, and the drug), A_c_ represents the absorbance of the control well (containing cells, culture medium, and CCK‐8 solution, but without the drug), and A_b_ represents the absorbance of the blank well (containing culture medium and CCK‐8 solution, but without cells or the drug).

### 2.5 Construction of the LPS‐RAW264.7 Inflammatory Cell Model and Determination of Pro‐Inflammatory Factor Levels

Logarithmic phase Raw264.7 cells were seeded in a 24‐well cell culture plate with a volume of 1 mL per well. The cell density was 5 × 10^5^ cells/mL, and they were cultured in DMEM medium containing 10% FBS. The experimental groups included a blank control group, an LPS‐treated group, and a drug‐treated group. When the cell density in the wells reached 70%–80%, the supernatant was discarded, and culture medium containing different concentrations of the drug was added to the wells. The plate was incubated for 4 h, followed by the addition of maintenance medium containing 10 μg/mL LPS for an additional 12 h of incubation. The cell culture medium was then collected and stored for further analysis. The experiment was conducted according to the instructions of the TNF‐α, PGE2, and COX‐2 ELISA Kits. The absorbance at a wavelength of 450 nm was measured using a microplate reader. Based on the standard curve generated, the levels of pro‐inflammatory factors TNF‐α [21], PGE2, and COX‐2 produced by Raw264.7 cells were calculated [22].

The blank control group did not receive LPS or any drug; the experimental groups included an LPS‐treated group, where each well‐received culture medium contained 10 μg/mL LPS; and a drug‐treated group, where different concentrations of the sample (with the maximum concentration being above 80% cell viability) were pretreated with the cells for 4 h [23], followed by the addition of maintenance medium containing 10 μg/mL LPS. Three biological replicates were conducted in this assay.

### 2.6 Determination of ROS Content in 3D Epidermal Model

For pretesting preparation, the model was transferred onto a six‐well plate, adding 2 mL of 3D full‐thickness skin model culture medium to each well. Then, the model was randomly allocated into the following groups: blank control (BC), negative control (NC), positive control (PC), and sample groups, with three replicates per group. Except for the blank control group (BC), the negative control (NC), positive control (VE), and sample groups were subjected to UVB radiation at a dose of 600 mJ/cm^2^. After the completion of radiation, the sample working solution was added to the liquid underneath the sample groups. Once the drug administration was complete, the model was transferred back to a culture dish and incubated in a CO_2_ incubator (37°C, 5% CO_2_) for 24 h. After the completion of the culture, the model's surface residue of the test substance was washed using a sterile PBS solution in a wash bottle. Any remaining liquid inside and outside the model was gently wiped away using a sterile cotton swab. Once the cleaning was done, the model was fixed with 4% paraformaldehyde for 24 h. After fixation, immunofluorescence detection was performed and images were captured using a fluorescence microscope. The images were analyzed using Image‐Pro Plus image processing software. Three biological replicates were conducted in this assay.

Relative IOD results can quantitatively reflect fluorescence intensity. The fundamental principle of IOD involves utilizing the computational capabilities of image processing software to aggregate the grayscale values across the region of interest (ROI), thereby deriving the total light intensity within this area. This total is then normalized by dividing it by the area encompassed by the ROI, yielding the average light intensity per unit area. The average light intensity from the ROI during the change period is subsequently subtracted from that of the control period, resulting in the quantification of staining variation in the skin tissue under investigation, which is represented as the IOD value. Results were plotted using GraphPad Prism, displaying the data as mean ± SEM. Consider *p* < 0.05 as indicating a significant difference and *p* < 0.01 as indicating a highly significant difference.

### 2.7 Skin Absorptive Capacity Test

Preparation of porcine skin in vitro: Immediately after sacrificing Bama pigs, the abdominal skin was carefully cut and separated from the subcutaneous fat layer and connective tissue. The skin was then rinsed with PBS and stored in a refrigerator at low temperature for future use. Prior to the experiment, the skin was thawed naturally, soaked in PBS for 30 min, dried with filter paper, and kept aside. The transdermal diffusion device used was a modified Franz diffusion apparatus, with an exposed skin area of 1.77 cm^2^ in the diffusion chamber and a receptor chamber volume of 8 mL.

The stir bar was placed into the receptor chamber. The porcine skin was cut into appropriate sizes and placed flat on the diffusion area. Wrapped it with two pieces of sealing film and secured it with clips. The stratum corneum faced the donor chamber, while the dermis faced the receptor chamber. 20 mg/mL EGCG, a 1‐mL EGCG aqueous solution wrapped in the EGCG, was taken and placed on the skin surface (donor chamber). Eight milliliters of acceptor fluid was added (the fluid level should be about 1 mm higher than the surface of the excised skin, ensuring stable contact between the liquid and the skin during the sampling process) and placed it in a 37°C constant temperature oven at a stirring speed of 300 r/min. The acceptor fluid: The selected acceptor fluids were as follows: PBS subcutaneous acceptor fluid sample: After 24 h of adding fluid to the donor chamber, 2 mL of acceptor fluid was taken in parallel three times. After sampling, the receptor chamber was replenished with an equal volume of temperature‐equilibrated 2 mL acceptor fluid and filtered through a 0.22‐μm microporous membrane for freezing at −20°C for further analysis. Intradermal sample: After 24 h of adding fluid to the donor chamber, the remaining liquid was removed from the donor chamber, and the skin was rinsed with 2 mL of PBS. The rinse solution was collected. The skin was ground with liquid nitrogen and then transferred to a 5‐mL centrifuge tube. It was then placed in a 50% methanol–water solution for 24 h. The sample solution was then collected [24]. Three biological replicates were conducted in this assay.

### 2.8 Determination of the Relative Content of ROS

Logarithmic growth phase HaCaT cells were seeded in a six‐well cell culture plate with 2.5 mL per well at a cell density of 5 × 10^4^ cells/mL, and cultured in DMEM medium containing 10% FBS. The groups were as follows: BC: PBS added without UVB irradiation; NC: PBS added with UVB irradiation; PC: Phosphatidylcholine added with UVB irradiation; EGCG and <EGCG>: EGCG and phosphatidylcholine‐encapsulated EGCG added with UVB irradiation. UVB radiation was set at a dose of 600 mJ/cm^2^. When the cell density reached 70%–80% in the wells of the plate, the cells were treated with different samples and incubated in a CO_2_ incubator for 24 h. After treatment with UVB, the cells were further incubated for 4–6 h. The culture medium in each well was removed using a pipette, and 200 μL of DCFH‐DA solution (dissolved in DMSO, final concentration 10 μM) was added to each well. The plate was incubated at 37°C for 15 min, and then the incubation solution of DCFH‐DA was aspirated. The cells were washed three times with serum‐free medium to remove DCFH‐DA that did not enter the cells, and then 100 μL of PBS solution was added to each well. The fluorescence intensity of the cells in each well was measured using a multifunctional microplate reader or flow cytometer under excitation wavelength of 485 nm and emission wavelength of 525 nm.

## 3 Results

### 3.1 Evaluation of Skin Absorptive Capacity for Phosphatidylcholine‐Encapsulated EGCG

**FIGURE 2:**
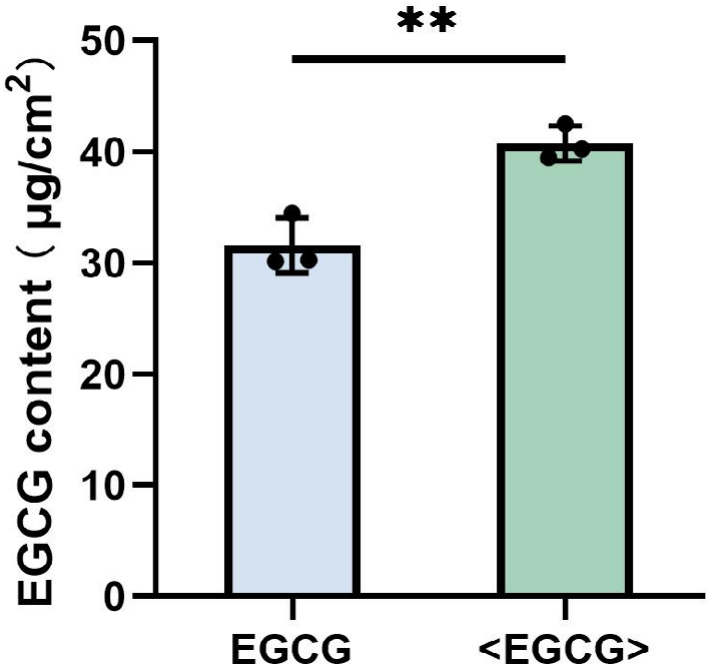
The contents of both EGCG and phosphatidylcholine‐encapsulated EGCG within the porcine skin. <EGCG>: phosphatidylcholine‐encapsulated EGCG. Samples were compared by *t‐*test, *represents the significant difference between groups, when *p* < 0.01, take **; when *p* < 0.05, take *.

The Bama porcine skin, approximately 1 mm thick, is a standard model for transdermal testing. Its close resemblance to human skin and exceptional quality make it the preferred model for the transdermal experiments conducted in this study. The results showed that neither EGCG nor phosphatidylcholine‐encapsulated EGCG were detected in the receiving solution. Additional analysis of the EGCG content in porcine skin indicated that the EGCG group had a concentration of 31.62 ± 2.01 μg/cm^2^, whereas the phosphatidylcholine‐encapsulated EGCG group exhibited a higher concentration of 40.76 ± 1.29 μg/cm^2^, demonstrating a noteworthy disparity (Figure 2). These results strongly suggested that both samples penetrated the skin, but none escaped into the surrounding solution. Moreover, the encapsulation of EGCG with phosphatidylcholine enhances the capacity of skin absorption.

### 3.2 Anti‐Inflammatory Activity of Phosphatidylcholine‐Encapsulated EGCG

**FIGURE 3:**
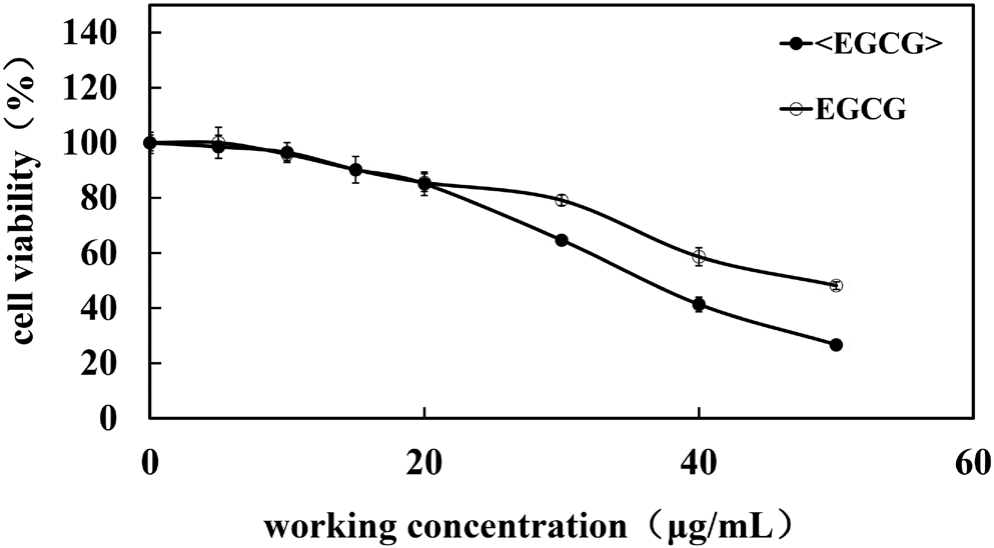
The effects of EGCG and <EGCG> on the cytotoxicity of HSF cells. <EGCG>: phosphatidylcholine‐encapsulated EGCG.

To investigate the anti‐inflammatory capabilities of phosphatidylcholine‐encapsulated EGCG, we employed LPS‐induced Raw264.7 cells as a model for cellular inflammation. Initially, we assessed the cytotoxic effects of both EGCG and phosphatidylcholine‐encapsulated EGCG on Raw264.7 cells. Concentrations that demonstrated cell viability above 80% were selected for subsequent anti‐inflammatory experiments. As shown in Figure 3, both EGCG and phosphatidylcholine‐encapsulated EGCG exhibited a gradual decrease in cell viability as the treatment concentration increased, indicating some degree of cytotoxicity. At a concentration of 20 μg/mL, the cell viability of phosphatidylcholine‐encapsulated EGCG was measured to be 85.09 ± 4.23%, while for EGCG alone, it was 85.56 ± 3.21%. The difference in cytotoxicity between the two formulations on Raw264.7 cells was not found to be statistically significant.

**FIGURE 4:**
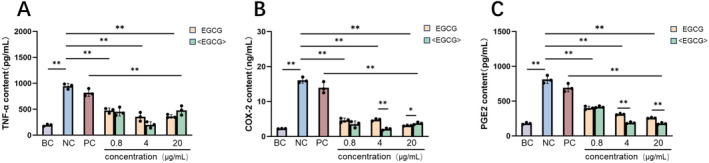
Results of different concentrations (μg/mL) of EGCG and phosphatidylcholine‐encapsulated EGCG inhibiting pro‐inflammatory cytokines. (A) EGCG and phosphatidylcholine‐encapsulated EGCG to pro‐inflammatory cytokine TNF‐α effect of secretion. (B) EGCG and phosphatidylcholine‐encapsulated EGCG to pro‐inflammatory cytokine COX‐2 effect of secretion. (C) EGCG and phosphatidylcholine‐encapsulated EGCG to pro‐inflammatory cytokine PGE2 effect of secretion. <EGCG>: phosphatidylcholine‐encapsulated EGCG. Analysis of variance was used for comparison among samples. *represents the significant difference between groups, when *p* < 0.01, take **; when *p* < 0.05, take *.

Taking into account the cytotoxicity findings, concentrations of 0.8 μg/mL, 4.0 μg/mL, and 20 μg/mL were chosen to assess the anti‐inflammatory efficacy of phosphatidylcholine‐encapsulated EGCG. Meanwhile, phosphatidylcholine was used as blank control group (PC). The cells were pretreated with phosphatidylcholine‐encapsulated EGCG, EGCG, and PC for 24 h, followed by induction of inflammation using 10 μg/mL LPS in Raw264.7 cells to trigger an inflammatory response [25]. Subsequently, the expression levels of pro‐inflammatory factors TNF‐α, COX‐2, and PGE2 were detected [26, 27, 28]. The results showed that treatment with EGCG and phosphatidylcholine‐encapsulated EGCG at concentrations of 0.8–20 μg/mL significantly reduced the secretion levels of pro‐inflammatory factors TNF‐α, PGE2, and COX‐2 compared to the LPS positive control group (Figure 4). At a concentration of 4 μg/mL, EGCG treatment reduced TNF‐α secretion by 62.48%, while phosphatidylcholine‐encapsulated EGCG reduced TNF‐α secretion by 78.84%. When the treatment concentration was increased to 20 μg/mL, there was no significant difference in the ability of EGCG and phosphatidylcholine‐encapsulated EGCG to inhibit TNF‐α secretion. This suggests that the increase in treatment concentration might have simultaneously increased the cytotoxicity of both EGCG and phosphatidylcholine‐encapsulated EGCG. Similar to the inhibitory effect on TNF‐α, treatment with encapsulated EGCG at a concentration of 4 μg/mL reduced COX‐2 secretion by 23.56% and showed a 24.82% reduction in the inhibition rate of PGE2 secretion compared to EGCG treatment alone. Additionally, as shown in Figure 4, there was no significant difference in the secretion of inflammatory factors between the phosphatidylcholine blank group (PC) and the model group (NC). However, a significant difference was observed between the PC group and the phosphatidylcholine‐coated EGCG group. This indicates that the phosphatidylcholine coating material itself did not significantly impact the results.

### 3.3 Analysis of the Antioxidant Protective Effects of Phosphatidylcholine‐Encapsulated EGCG

**FIGURE 5:**
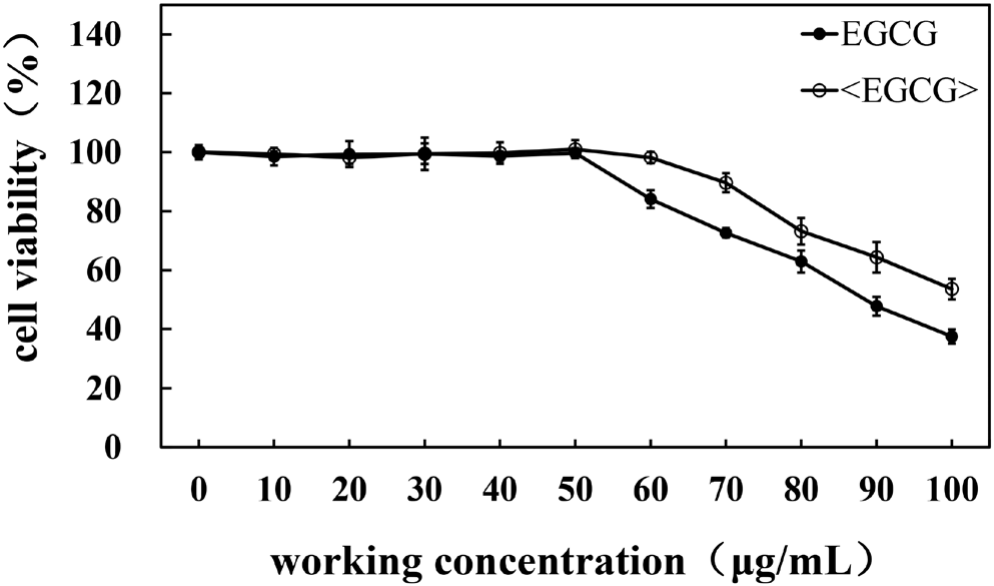
The effects of EGCG and <EGCG> on the cytotoxicity of HaCaT cells. <EGCG>: phosphatidylcholine‐encapsulated EGCG.

To assess the antioxidant capacity of EGCG and phosphatidylcholine‐encapsulated EGCG, the first step was to evaluate their cytotoxic effects on skin cells. HaCaT cells, a type of keratinocyte, were selected as a cell model, and its cell viability was assessed using the CCK‐8 assay. The experimental results showed that at a concentration of 10 μg/mL, the phosphatidylcholine‐encapsulated EGCG treatment resulted in a cell viability of 84.41 ± 4.61% in HaCaT cells, significantly higher than the EGCG group's viability of 70.30 ± 1.40% (Figure 5). At concentrations higher than 10 μg/mL, the viability of HaCaT cells treated with phosphatidylcholine‐encapsulated EGCG was higher than that of cells treated with EGCG alone, indicating that phosphatidylcholine‐encapsulated EGCG exhibits lower toxicity to HaCaT cells compared to EGCG alone. Based on the results of the anti‐inflammatory experiments above, it was evident that 4 μg/mL of phosphatidylcholine‐encapsulated EGCG exhibited the most effective cell inflammation reduction. Taking into account the cell toxicity results, a concentration of 4 μg/mL of EGCG and phosphatidylcholine‐encapsulated EGCG were selected for the evaluation of antioxidant activity.

**FIGURE 6:**
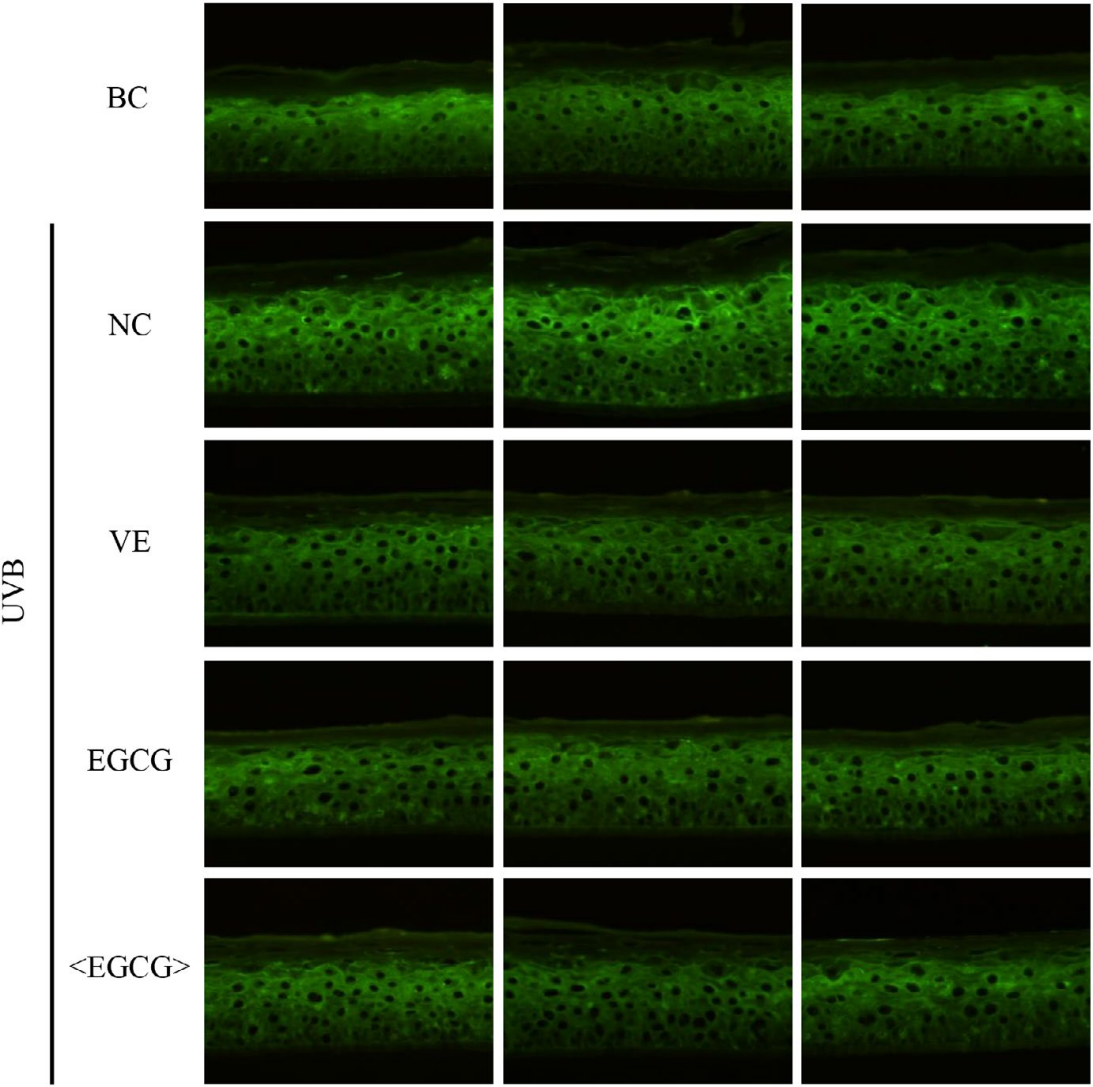
Fluorescence detection results of ROS levels in UVB‐irradiated 3D epidermal models. BC: blank control; NC: negative control; VE: positive control; <EGCG>: phosphatidylcholine‐encapsulated EGCG.

**FIGURE 7:**
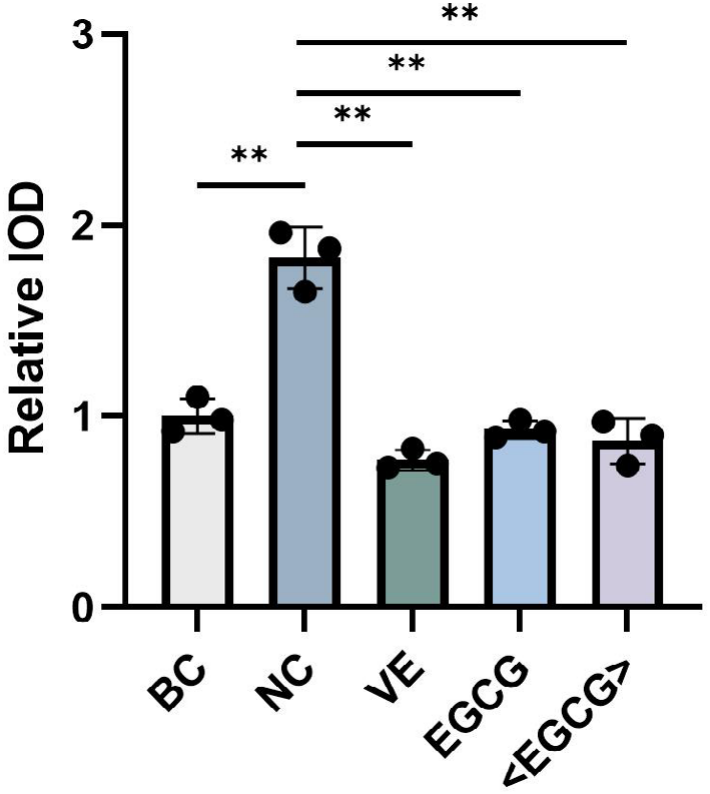
The relative IOD value of ROS in 3D epidermal models treated with EGCG and phosphatidylcholine‐encapsulated EGCG. BC: blank control; NC: negative control; VE: positive control; <EGCG>: phosphatidylcholine‐encapsulated EGCG. *p* < 0.05 is denoted as “*”, and *p* < 0.01 is denoted as “**.”

In this study, an evaluation model for antioxidant activity was developed using UVB (600 mJ/cm^2^) treated 3D epidermal model Epikutis. EpiKutis features a layered structure that closely mirrors the natural composition of skin and exhibits a high degree of histological similarity to human skin. It includes the stratum corneum, stratum granulosum, stratum spinosum, and stratum basale. When performing antioxidant assays, employing EpiKutis skin models allows for a more authentic simulation of real skin conditions, resulting in more scientifically valid outcomes. Consequently, this model is utilized in the antioxidant test in this study. The fluorescence probe was utilized to detect the levels of reactive oxygen species (ROS). The experimental results, as shown in Figure 6, indicate that the fluorescence intensity in HaCaT cells treated with EGCG and phosphatidylcholine‐encapsulated EGCG was significantly lower compared to the negative control group (NC) without any treatment. The data analysis results demonstrated that the phosphatidylcholine‐encapsulated EGCG group and the EGCG group exhibited a respective decrease of 0.96 and 0.9 in relative integrated optical density (IOD) compared to the negative control group (Figure 7). This indicated that both phosphatidylcholine‐encapsulated EGCG and EGCG had significant ROS scavenging ability, although the difference between them is not significant.

**FIGURE 8:**
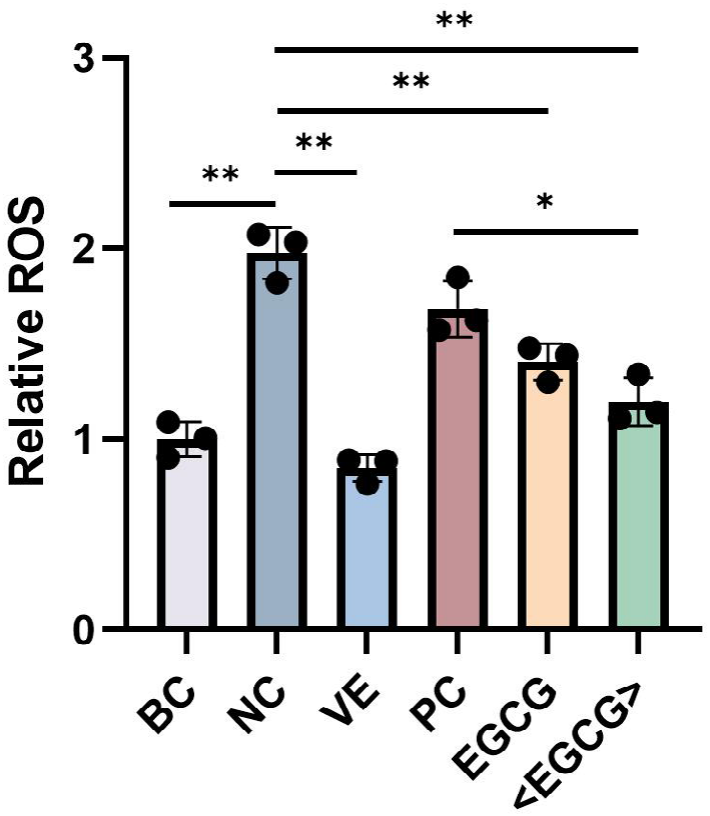
The effect of EGCG and phosphatidylcholine‐encapsulated EGCG on intracellular ROS scavenging. BC: blank control; NC: negative control; VE: positive control; <EGCG>: phosphatidylcholine‐encapsulated EGCG. *p* < 0.05 is denoted as “*”, and *p* < 0.01 is denoted as “**.”

To further verify the above results, ROS scavenging tests were conducted for each sample at the cellular level. Blank phosphatidylcholine was added as a control (PC group). The findings indicated that, compared to the model group (NC group), both the EGCG group and the encapsulated EGCG group significantly reduced intracellular ROS levels, demonstrating strong antioxidant capacity. The PC group showed no significant difference from the model group, indicating that the encapsulating material itself did not exhibit significant antioxidant function. These results aligned well with those in Figures 6 and 7, further confirming the significant antioxidant capacity of both EGCG and phosphatidylcholine‐encapsulated EGCG (Figure 8).

## 4 Discussion

Utilizing amphiphilic phosphatidylcholine as encapsulation material is a commonly employed strategy for improving cosmetic ingredients [29]. In this study, the skin absorbency, the anti‐inflammatory and antioxidant effects of EGCG, and phosphatidylcholine‐encapsulated EGCG were investigated and horizontally compared. The research results demonstrated that the skin absorption capacity of phosphatidylcholine‐encapsulated EGCG was 28.91% higher compared to EGCG alone, indicating that the use of phosphatidylcholine encapsulation improves the skin absorption capacity of EGCG. Compared to EGCG alone, phosphatidylcholine‐encapsulated EGCG significantly inhibits the secretion of pro‐inflammatory factors TNF‐α, COX‐2, and PGE2 in LPS‐induced macrophages. This expands its application value as a cosmetic ingredient in terms of absorption capacity and functionality.

EGCG is a catechin compound with a hydroxyl group at position 8 and a four‐ring structure, exhibiting strong hydrophilicity. However, the skin's barrier function, responsible for its water permeability, is primarily attributed to the extracellular matrix between corneocytes, which is composed of lipid‐rich lamellar arrays [30]. Cell membranes are composed of a phospholipid bilayer, which serves as the basic framework and is embedded with various protein and carbohydrate molecules. The absorption capacity of lipophilic small molecules is much higher than that of hydrophilic small molecules, indicating that cell membranes might have a lower absorption rate for highly hydrophilic substances like EGCG. When encapsulated with phospholipid polymers composed of amphiphilic phosphatidylcholine, the hydroxyl groups of EGCG were hidden and replaced by phosphatidylcholine groups. This not only enhanced the lipophilicity of EGCG but also increased its affinity for the phospholipid bilayer of cell membranes, making it easier to be absorbed by cells through passive transport or endocytosis.

TNF‐α is one of the main factors secreted by macrophages and can also stimulate the secretion of other inflammatory factors, such as COX‐2 and PGE2 [31]. Studies have shown that EGCG inhibits the expression and secretion of inflammatory factors in different types of cells by upregulating peroxisome proliferator‐activated receptor gamma (PPARγ) and downregulating p38 mitogen‐activated protein kinase (MAPK) and nuclear factor‐kappa B (NF‐κB) [32]. Our results indicated that the phosphatidylcholine‐encapsulated EGCG exhibited a higher skin absorption capacity. This might be one of the reasons why the phosphatidylcholine‐encapsulated EGCG had stronger inhibitory effects on pro‐inflammatory factors compared to EGCG alone. Another study has shown that phosphatidylcholine can inhibit the expression of the p38 pathway [33]. Therefore, it is possible that the phosphatidylcholine‐encapsulated EGCG had a better inhibitory effect on the secretion of pro‐inflammatory factors due to the combined effects of enhanced absorption and the action of phosphatidylcholine.

This study was dedicated to examining the anti‐inflammatory and antioxidant properties of EGCG encapsulated with phosphatidylcholine, highlighting its distinct efficacy relative to EGCG. Additionally, its robust scavenging capability for ROS remained on par with that of EGCG. Despite this, the current evaluation metrics are limited. Subsequent research should endeavor to explore a broader spectrum of aspects, including potential significant functions in whitening, antiwrinkle, soothing, antisensitivity, and beyond. Such an extensive investigation is essential to achieve a comprehensive understanding of the efficacy of encapsulated EGCG as a versatile cosmetic raw material. Furthermore, it is important to acknowledge that the data derived from this study are exclusively the outcomes of in vitro experiments. Moving forward, it will be imperative to conduct subsequent in vivo experiments, building upon these foundational research findings, to validate the rigor and practicality of the research conclusions.

## Author Contributions

Conceptualization: Minjia Yuan, Hang Tie and Liang Xu. Methodology: Lili Hu and Cuicui Zhu. Validation: Qi Li and Hang Tie. Writing – original draft preparation: Minjia Yuan, Liang Xu. Writing – review and editing: Haihua Ruan Tao Wu and Hongyang Zhang. Supervision: Liang Xu and Hang Tie. All authors have read and agreed to the published version of the manuscript.

## Conflicts of Interest

The authors declare no conflicts of interest.

## Supporting information


**Figure S1.** Schematic illustration of the EGCG model encapsulated in phosphatidylcholine.

## Data Availability

The data that support the findings of this study are available from the corresponding author upon reasonable request.
