# Supplementary figures and images for: Comparison and Assessment of Anti‐Inflammatory and Antioxidant Capacity Between EGCG and Phosphatidylcholine‐Encapsulated EGCG
Source: J Cosmet Dermatol. 2024 Oct 31;24(1):e16628. doi: 10.1111/jocd.16628 (PMC11743248; doi:10.1111/jocd.16628)

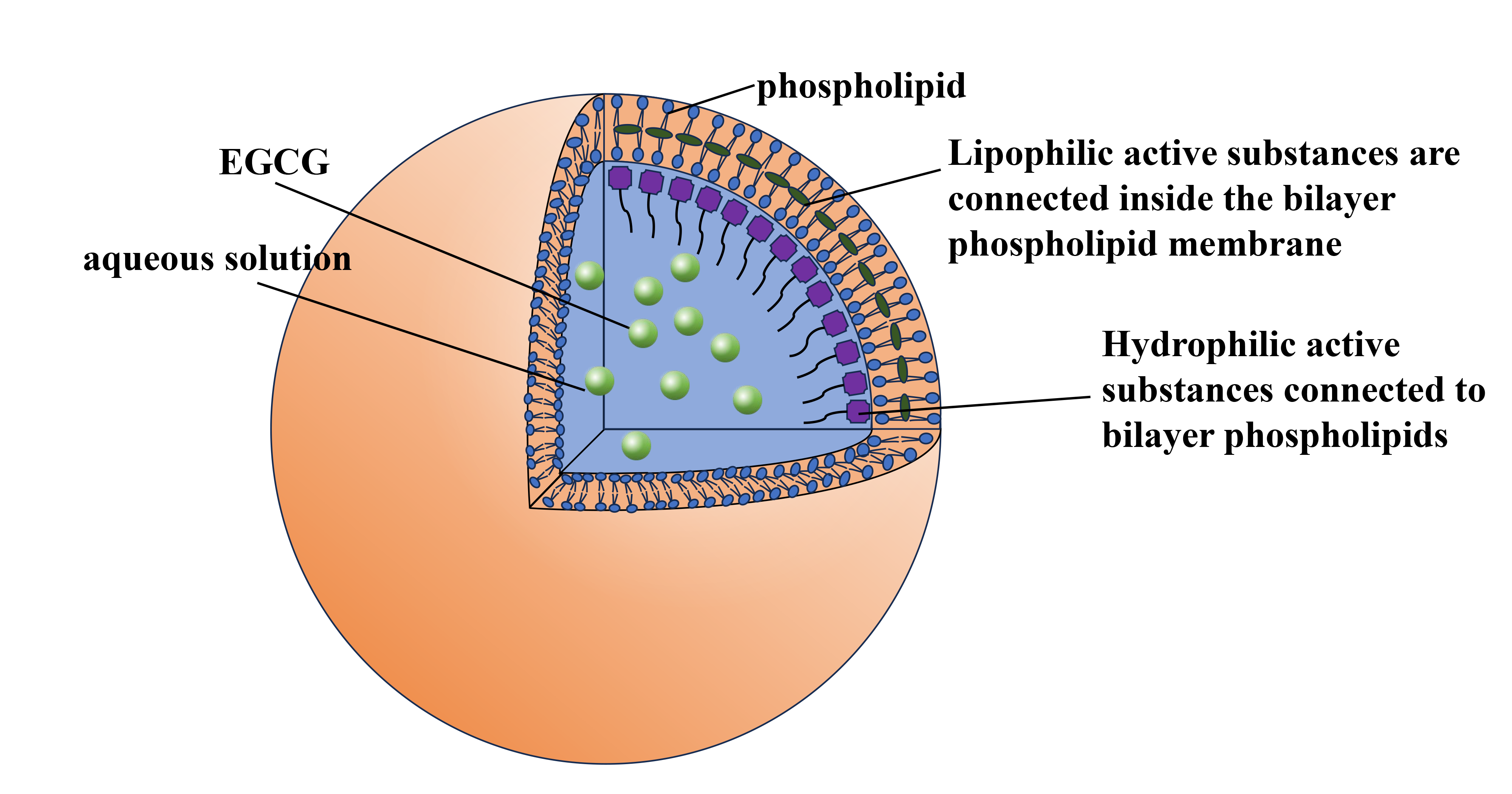

Supplement: Supplementary file 1 — Figure S1. Schematic illustration of the EGCG model encapsulated in phosphatidylcholine. [file JOCD-24-e16628-s001.tif]
